# PPARγ activation improved learning and memory and attenuated oxidative stress in the hippocampus and cortex of aged rats

**DOI:** 10.14814/phy2.15538

**Published:** 2022-12-21

**Authors:** Farimah Beheshti, Masoumeh Gholami, Zahra Ghane, Seyedeh Elnaz Nazari, Maryam Salari, Sadegh Shabab, Mahmoud Hosseini

**Affiliations:** ^1^ Neuroscience Research Center Torbat Heydariyeh University of Medical Sciences Torbat Heydariyeh Iran; ^2^ Department of Physiology, School of Paramedical Sciences Torbat Heydariyeh University of Medical Sciences Torbat Heydariyeh Iran; ^3^ Department of Physiology, Faculty of Medicine Arak University of Medical Sciences Arak Iran; ^4^ Psychiatry and Behavioral Sciences Research Center Mashhad University of Medical Sciences Mashhad Iran; ^5^ Applied Biomedical Research Center Mashhad University of Medical Sciences Mashhad Iran; ^6^ Neuroscience Research Center Mashhad University of Medical Sciences Mashhad Iran; ^7^ Department of Physiology, School of Medicine Mashhad University of Medical Sciences Mashhad Iran

**Keywords:** aging, learning and memory, oxidative stress, pioglitazone, PPARγ

## Abstract

Oxidative stress has an important role in brain aging and its consequences include cognitive decline and physiological disorders. Peroxisome proliferator‐activated receptor‐γ (PPARγ) activation has been suggested to decrease oxidative stress. In the current research, the effect of PPARγ activation by pioglitazone(Pio) on learning, memory and oxidative stress was evaluated in aged rats. The rats were divided into five groups. In the Control group, vehicle (saline‐diluted dimethyl sulfoxide (DMSO)) and saline were injected instead of Pio and scopolamine (Sco), respectively. In the Sco group, the vehicle was injected instead of Pio and the rats were injected by Sco 30 min before the behavioral tests. In the Sco‐Pio 10, Sco‐Pio 20, and Sco‐Pio 30 groups, 10, 20, and 30 mg/kg Pio was injected and finally, the rats were injected with Sco 30 min before the behavioral tests. Morris water mater maze(MWM) and passive avoidance(PA) tests were carried out, and finally, the hippocampus and cortex were removed for biochemical assessments. The results showed that the highest dose of Pio decreased the traveling time and distance during 5 days of learning and increased the time and distance in the target area on the probe day of MWM. The highest dose of Pio also prolonged the delay time for entering the dark and total time spent in the light while decreasing the total time spent in and the number of entries into the dark in PA test. Pio especially, in the medium and highest doses, decreased MDA while increasing thiol, superoxide dismutase, and catalase in the hippocampus and cortex. It is concluded that PPARγ activation by Pio as an agonist improved learning and memory in aged rats probably by attenuating oxidative stress in the hippocampus and cortex.

## Graphical abstract

PPARγ activation by Pioglitazone as an agonist improved learning and memory in aged rats probably by attenuating oxidative stress in the hippocampus and cortex.

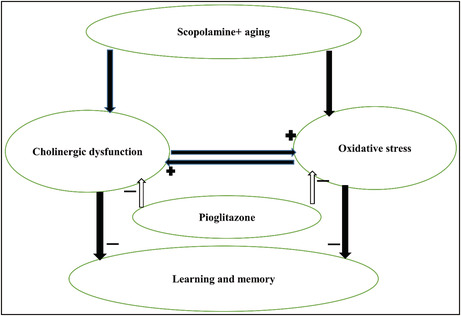

## 1 INTRODUCTION

Aging is a natural process that causes cognitive decline and physiological disorders (Jia et al., 2016; Oh & Nam, 2019), which is associated with many diseases such as Alzheimer's disease(AD), Parkinson's disease, and Huntington's disease (Guyot et al., 2020; Horvath et al., 2016). Recorded data shows that 11 percent of the world's population is over the age of 60, which is projected to increase to more than one billion over the next 10 years (Baierle et al., 2015; Chen et al., 2017). Loss of memory is a typical feature of natural aging (Larrayoz et al., 2017) which is closely related to age‐related brain changes (Souza et al., 2015). In older communities, cognitive processes and in particular, long‐term memory development, preservation, and regeneration are disrupted (Koen & Yonelinas, 2014). Brain aging is also a poorly understood complex condition correlated with the deterioration of executive functioning along with motor and sensory problems (Casu et al., 2002). In aging conditions neurotransmitter systems are also disturbed, among them cholinergic system dysfunction is noticeable (Schliebs & Arendt, 2011). Cholinergic dysfunction induced by scopolamine (Sco) has been repeatedly used as a well‐known animal model to examine the effects of the drugs on learning and memory or to challenge the mechanism(s) responsible for learning and memory impairments (Azizi‐Malekabadi et al., 2012; Chen & Yeong, 2020; Jamialahmadi et al., 2013; Tang, 2019). This agent acts as a muscarinic receptor antagonist to impair learning and memory and to produce an animal model which mimics AD (Azizi‐Malekabadi et al., 2012; Chen & Yeong, 2020; Jamialahmadi et al., 2013; Tang, 2019). It is well known that Sco induced learning and memory impairment is accompanied with an oxidative stress status in the brain (Ghasemi & Moradzadeh, 2019; Hosseini et al., 2015; Tang, 2019).

One of the most common causes of memory loss in the elderly and responsible for the events that lead to aging is inflammation and oxidative stress (Picca et al., 2022).Oxidative stress is considered as an imbalance between reactive oxygen species (ROS) and reactive nitrogen species (RNS) and weak antioxidant defenses such as reduced enzyme activity such as superoxide dismutase (SOD) and glutathione peroxidase (GSH‐Px) (Baierle et al., 2015). Excessive accumulation of ROS and oxidative stress damage lipids, proteins, and cellular DNA disrupting cellular function and eventually leading to the death of neuronal cells (Ali et al., 2015). Oxidative stress is suggested to have an important role in brain aging and age‐related cognitive impairments (Tönnies & Trushina, 2017). Therefore, antioxidant drugs, vitamins, and natural products are considered to have improving effects on age‐related cognitive dysfunction (Giudetti et al., 2018).

Peroxisome proliferator‐activated receptors (PPARs) are ligand‐dependent transcription factors. Activation of the PPARγ subtype is known to increase insulin sensitization, and modulate glucose and lipid metabolism. Pioglitazone(Pio) is a thiazoledinedione (TZD) and a selective PPARγ agonist (Sood et al., 2000). Recently, the focus on PPARγ agonists has intensified, as their novel biological roles have emerged, particularly for their therapeutic potential in neurodegenerative disorders, such as AD (Nicolakakis & Hamel, 2010). The effects of PPARγ agonists including Pio on learning and memory has been reported (Almasi‐Nasrabadi et al., 2014; Gupta & Gupta, 2012; Xiang et al., 2012); however, the effects of Pio on learning and memory impairments induced by Sco and oxidative stress indicators in the brain tissues of aged rats has not been addressed. Considering the roles of oxidative stress in age‐related learning and memory impairment, and considering the antioxidant role of PPARγ agonists, it was hypothesized that PPARγ activating by Pio may improve learning and memory in aging conditions. Therefore, this study was undertaken to investigate the effects of PPARγ activation by Pio on learning and memory impairment induced by Sco, and hippocampal and cortical tissue oxidative stress in aged rats.

## 2 MATERIALS AND METHODS

### 2.1 Animals and drugs

Thirty‐five aged (28–29 months, 350–380 g weight) male Wistar rats were allocated into five groups of seven in each group. The animals were kept in the animal lab of Mashhad University of Medical Sciences. The animals were maintained under standard conditions with a 12‐hr light/dark cycle and average temperature (22–24°C) with free reach to laboratory food and water in the animal house (Lights on at 6:00 am). All experiments were performed under the Mashhad University of Medical Sciences Ethics Committee (IR.MUMS.MEDICAL.REC.1398.313).

The grouping was as follows:

Control group; the old rats received 1 ml/kg saline diluted dimethyl sulfoxide (DMSO) as a vehicle instead of Pio intraperitoneally (IP) and normal saline injection (IP) instead of Sco. Sco group; the vehicle was injected instead of Pio for two weeks but treated by Sco (2 mg/kg, Sigma Chemical Co) 30 min before each behavioral test during the third week (Mohammadpour et al., 2015). Sco‐Pio 10, Sco‐Pio 20, and Sco‐Pio 30 groups; the rats were treated daily with 10g, 20, or 30 mg/kg (Baghcheghi et al., 2016; Baghcheghi et al., 2019; Beheshti et al., 2019). Pio dissolved in saline diluted DMSO (final concentration 10%) for 3 weeks, and then they were also injected by Sco (2 mg/kg, i.p.) 30 min before the behavioral tests.

**FIGURE 1:**
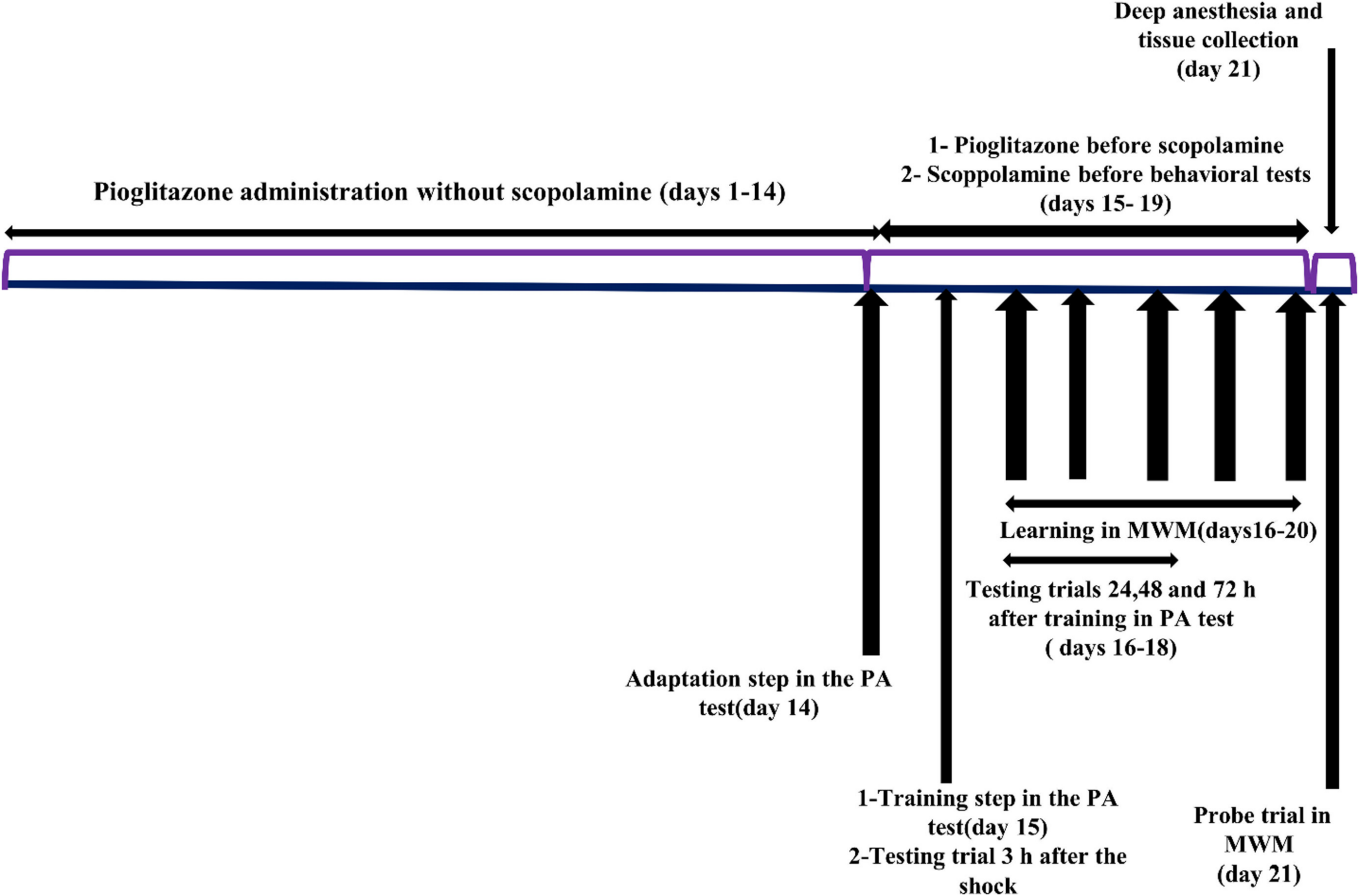
A graphical timeline demonstrating the sequence of experimental protocols. MWM, Morris water maze; PA, Passive avoidance test.

The experiments were done for 3 weeks. The groups were treated for two weeks as described above. In the third week, Pio or vehicle was daily injected 30 min before Sco, and Sco was injected 30 min before doing the behavioral tests. The behavioral tests included Morris water maze (MWM) and passive avoidance (PA) and they were done between 09:00 am and 03:00 pm. A graphical timeline demonstrating the sequence of experimental protocols is shown in Figure 1.

### 2.2 Behavioral tests

Spatial memory and learning measurement was done using the MWM study. The apparatus was including a pool of 136 cm in diameter and 60 cm in height. The pool was filled with water (23–25°C) up to 30 cm. Besides, the pool itself was hypothetically divided into four quarters. There was also a circular and transparent platform with a diameter of 10 cm and a height of 28 cm which was located in the pool.

During the first five days of the experiment, each rat was released into the pool four times from different positions (north (N), south (S), east (E), and west (W)). The software randomly determined the rat's release positions. All movements and travel routes were recorded by a camera and transferred to the computer via an interface. The distance (path length) and delay time to reach the platform were extracted from the software's processed films. Each rat was given 60 s to search the platform in the pool. If the rat considered the platform for this time or less, it was allowed to stay on it for 15 s. If the rat could not find the platform for 60 s, it was directed to the platform and remained on it for 15 s. On day 6, a probe test was performed to analyze the rats to remember the platform's location. In the probe test, the platform was removed, and each rat was released into the pond and given 60 s for searching the platform. The time elapsed in the target quadrant and the traveled distance in this area were reported for comparison between groups (Asghari et al., 2022; Beheshti, Karimi, et al., 2017).

Non‐hippocampal‐dependent learning and memory were evaluated using the PA test. The device was divided by a guillotine door into light and dark places. The animals were first placed into the equipment to move freely between the two spaces for five minutes when the guillotine door was opened. An electric shock (2 mA, 2 s) was applied to the foot of the rats in an acquisition experiment when they entered the darkroom. After 3, 24, 48, and 72 h, the animals were placed in the light chamber, and the delay in dark composition, time of light and darkness, and frequency of entering into the dark part were recorded (Asghari et al., 2022; Beheshti, Karimi, et al., 2017).

### 2.3 Biochemical assessments

The animals were anesthetized by urethane (1.4 g/kg) and sacrificed after the last day of the behavioral study. The hippocampal tissues and all parts of the cortex were then removed. Total thiol and malondialdehyde (MDA) concentration and superoxide dismutase (SOD), and catalase (CAT) activities were detected in cortical and hippocampal tissues.

As lipid peroxidation index, MDA concentration in the hippocampal and cortical tissues was measured according to a previously described protocol (Beheshti, Karimi, et al., 2017; Mansouri et al., 2021). MDA reacts with thiobarbituric acid (TBA) to form a red complex. The absorbance was read at 535 nm. Besides, total thiol concentration was determined in both the hippocampus and cortex. In this procedure, the reaction between DTNB (2,2′‐dinitro‐5,5′‐dithiol benzoic acid) and thiol groups forms a yellow complex. Absorbance was read at 412 nm (Beheshti, Karimi, et al., 2017; Mansouri et al., 2021).

The method for both SOD and CAT activity was as previously reported (Beheshti, Karimi, et al., 2017). The SOD activity was measured using the Madesh and Balasubramanian processes (Madesh & Balasubramanian, 1998; Mansouri et al., 2021). The SOD activity was measured at 570 nm according to a colorimetric technique. One unit of SOD was equal to the amount of enzyme that should be inhibited by 50% of the MTT reduction rate. The Aebi method was used to measure CAT activity using hydrogen peroxide (30 mM) as a substrate (Aebi et al., 1976; Mansouri et al., 2021).

### 2.4 Statistical Analysis

We provided the data as means ± SEM. Repeated measures ANOVA, one‐way ANOVA, and Tukey's post hoc tests were utilized to evaluate the behavioral and biochemical data. To evaluate data, SPSS software (version 26 Chicago, IL) was used. Differences were considered statistically significant when *p* < 0.05.

## 3 RESULTS

### 3.1 PPARγ activation improved the performance of the aged rats in MWM

**FIGURE 2:**
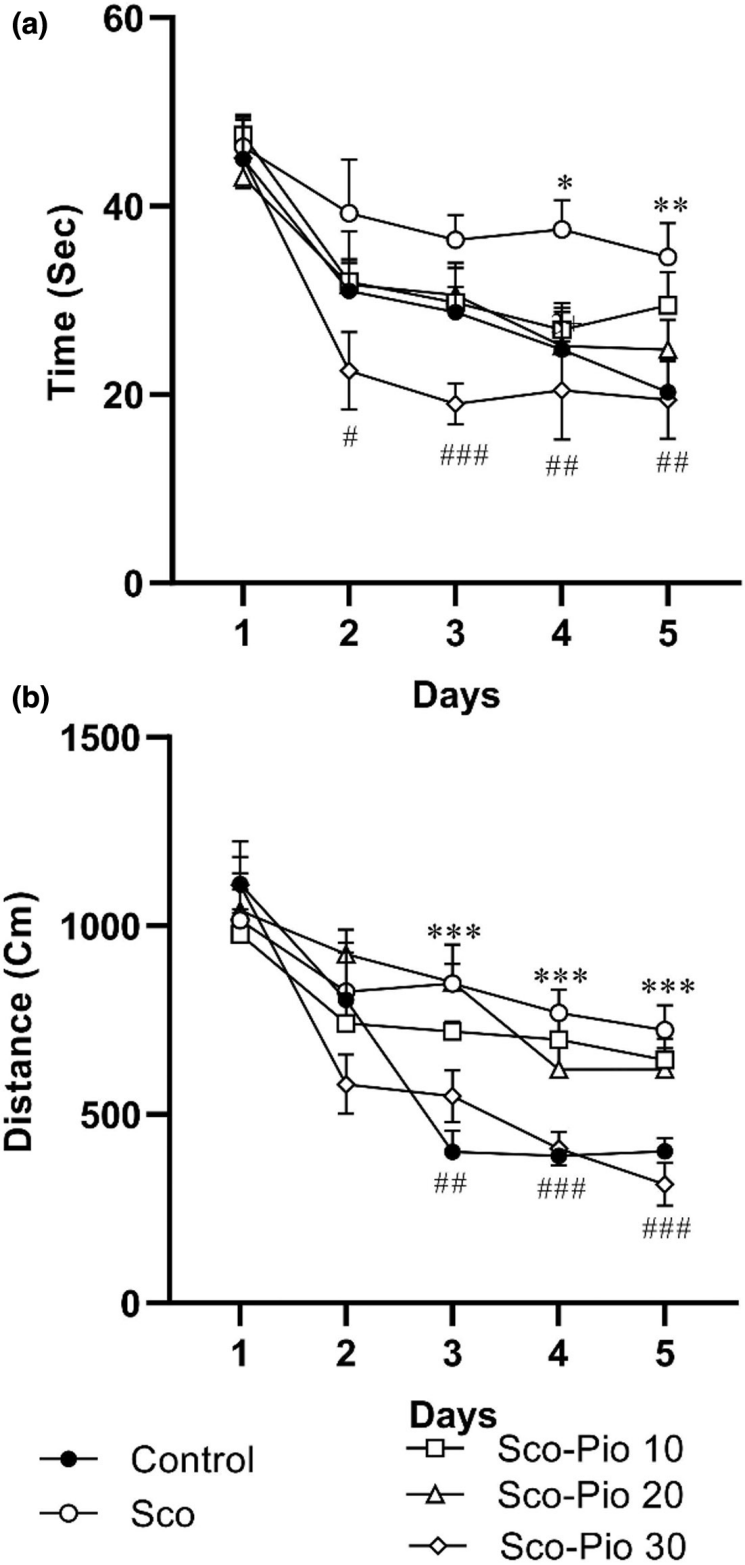
The results of traveling time (a) and distance (b) in the learning phase of the Morris water maze test. *p < 0.05, **p < 0.01, and ***p < 0.001 present the difference between Sco group and Control group, ^#^ *p* < 0.05, ^##^ *p* < 0.01, and ^###^ *p* < 0.001 present the difference between Sco‐Pio 30 group and Sco group. The data are presented as mean ± SEM (*n* = 7 per group). Sco: scopolamine, Sco‐Pio 10: scopolamine–pioglitazone 10 mg/kg, Sco‐Pio 20: scopolamine–pioglitazone 20 mg/kg, Sco‐Pio 30: scopolamine–pioglitazone 30 mg/kg.

The results indicated that the time and path to reach the hidden platform in the Sco group were significantly higher than in the Control group (*p* < 0.05 to *p* < 0.001). Pretreatment with 30 mg/kg Pio decreased the escape latency and traveled distance compared to the Sco group (*p* < 0.05 to *p* < 0.001). There was no significant difference between the rats pre‐treated by 10 and 20 mg/kg Pio and Sco group in the elapsed time and traveled distance to reach the platform (Figure 2a,b).

**FIGURE 3:**
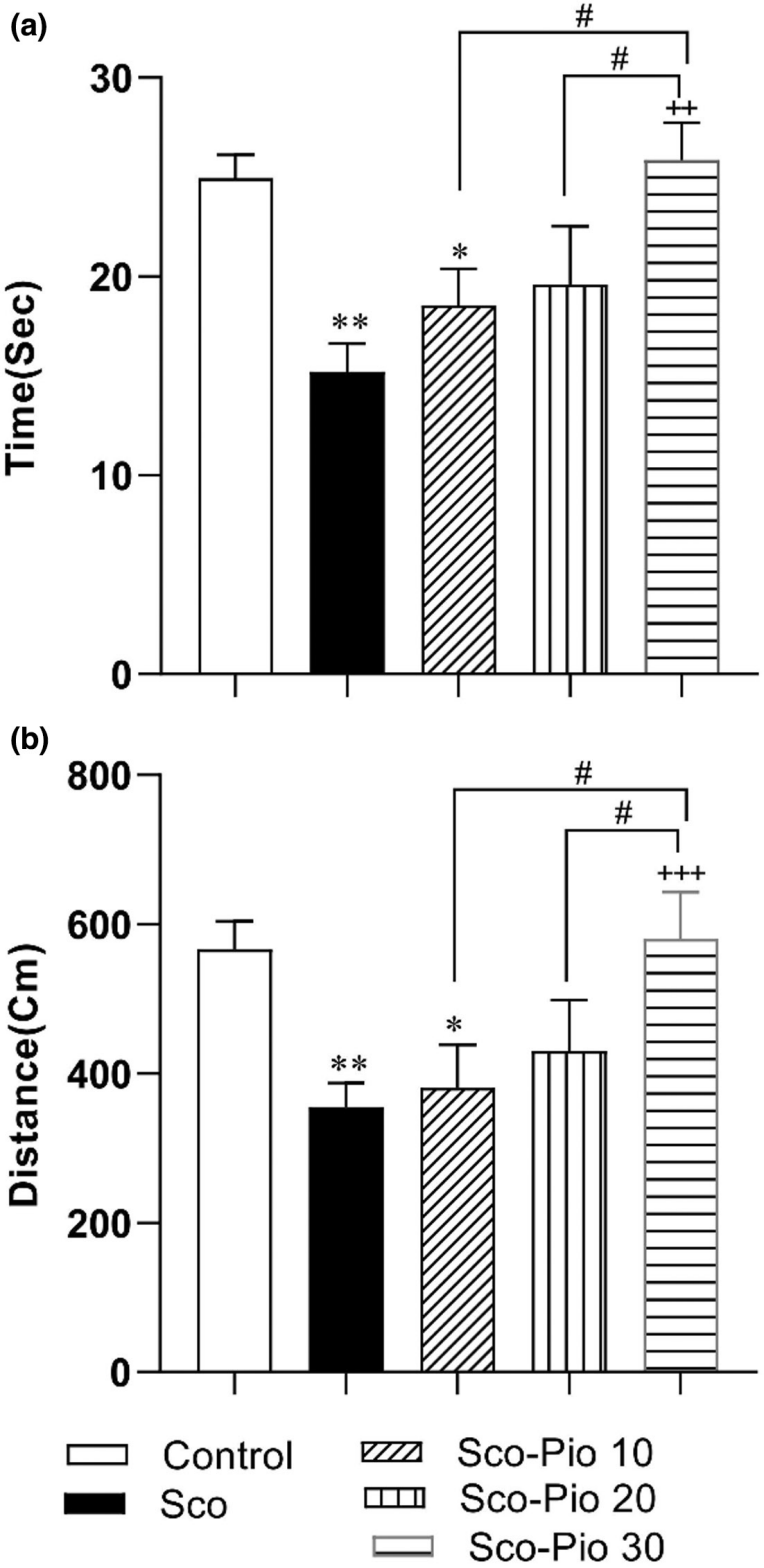
The results of traveling time (a) and distance (b) in the target area trial on the sixth day of the Morris water maze test in which the probe trial was done. *p < 0.05 and **p < 0.01 present the difference between other groups and Control group, ^++^ *p* < 0.01 and ^+++^ *p* < 0.001 present the difference between other groups and Sco group, ^#^ *p* < 0.05 presents the difference between other Sco‐Pio 10, Sco‐Pio 20, and Sco‐Pio 30 groups. The data are presented as mean ± SEM (*n* = 7 per group). Sco: scopolamine, Sco‐Pio 10: scopolamine–pioglitazone 10 mg/kg, Sco‐Pio 20: scopolamine–pioglitazone 20 mg/kg, Sco‐Pio 30: scopolamine–pioglitazone 30 mg/kg.

On probe day, the results showed that animals in the Sco group spent less time and traveled a shorter distance in the target quadrant than the Control group (*p* < 0.01). Administration of Pio at the dose of 30 mg/kg, improved the spatial memory of rats and the rats spent a longer time and traveled longer distance in the target quadrant than the rats of Sco group (*p* < 0.01 for both). Additionally, none of 10 and 20 mg/kg didn't change the traveling time and distance in the target part of MWM (Figure 3a,b). The result also showed that the rats of Sco‐ Pio 30 group spent longer time and traveled longer distance in the target area of MWM than the rats of both Sco‐ Pio 31 and Sco‐ Pio 20 groups (*p* < 0.01 for both).

### 3.2 PPARγ activation improved performance of the aged rats in PA test

**FIGURE 4:**
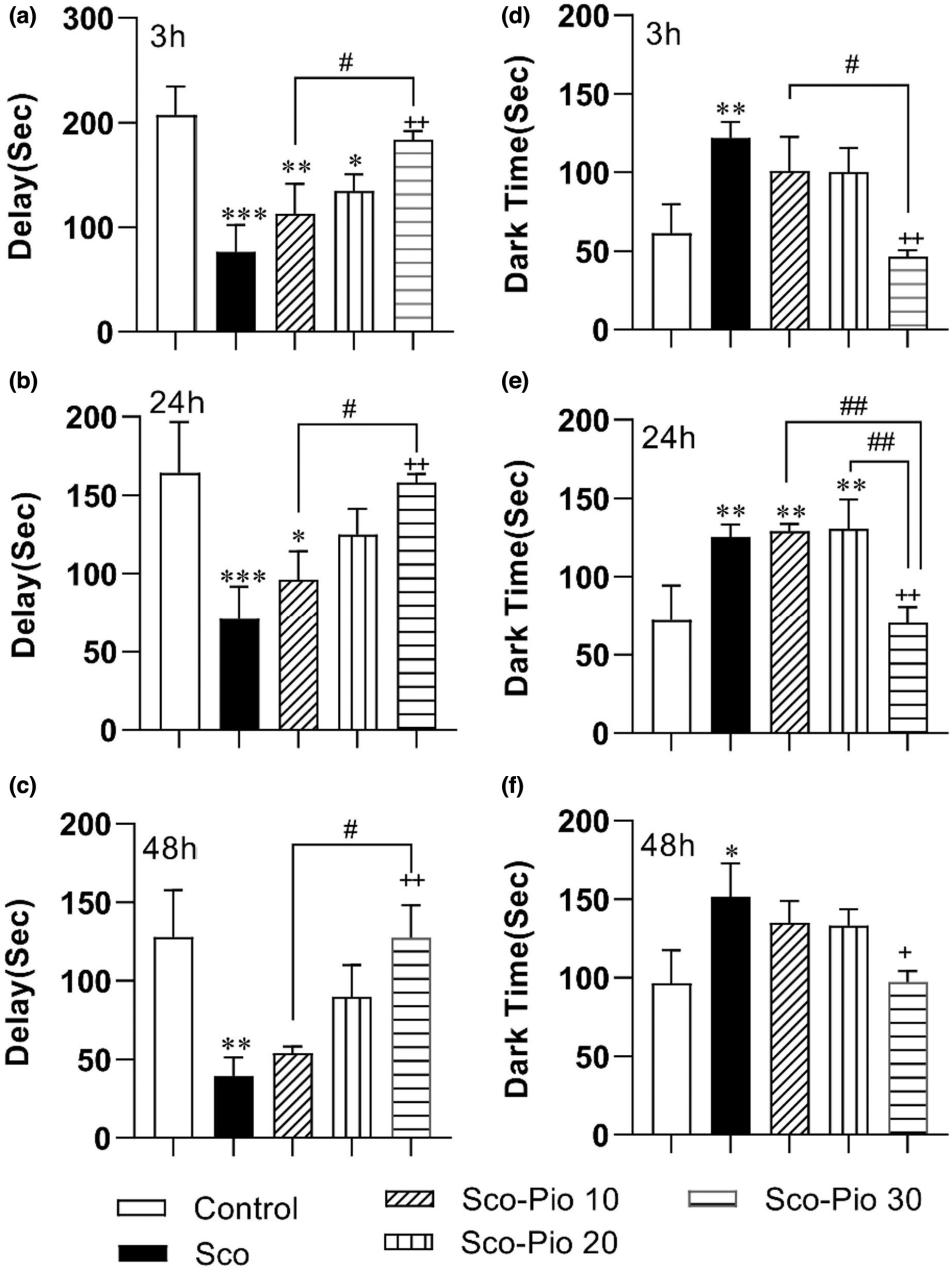
The results of delay for entering the dark (a–c) and the total dark time (d–f) in the passive avoidance test. *p < 0.05, **p < 0.01, and ***p < 0.001 present the difference between other groups and Control group, ^+^ *p* < 0.05 and ^++^ *p* < 0.01 present the difference between other groups and the Sco group, ^#^ *p* < 0.05 and ^##^ *p* < 0.01 present the difference between Sco‐Pio 10, Sco‐Pio 20, and Sco‐Pio 30 groups. The data are presented as mean ± SEM (*n* = 7 per group). Sco: scopolamine, Sco‐Pio 10: scopolamine–pioglitazone 10 mg/kg, Sco‐Pio 20: scopolamine–pioglitazone 20 mg/kg, Sco‐Pio 30: scopolamine–pioglitazone 30 mg/kg.

However, by comparing the delay time to enter the dark chamber after administering the shock, it was found that the rats of Sco group had a shorter time to enter the dark chamber at all 3, 24, and 48 h after the shock compared to the Control group (*p* < 0.01 to *p* < 0.001). The results also showed that 30 mg/kg Pio increased delay time compared to the Sco group at all times, including 3, 24, and 48 h after the shock, and protected against harmful effects of Sco (*p* < 0.01 at all times; Figure 3a). None of 10 and 20 mg/kg was not effective on the delay time. In addition, the delay time for entering the dark in the rats treated with 30 mg/kg Pio was longer than the ones treated with 10 mg/kg (*p* < 0.05 at all times) (Figure 4a).

The results also showed that the dark time in the Sco group was longer in the dark than that in the Control group at all 3, 24 ad 48 h after the shock (*p* < 0.05 to *p* < 0.01). Treatment by the highest dose (30 mg/kg) of Pio was able to shorten the dark time at all 3, 24, and 48 h post‐shock time (*p* < 0.01 at all times) but the lowest and the medium doses were not effective (Figure 4b). The dark time in the Sco‐Pio 30 was shorter than that in Sco‐Pio 10 group at 3 and 24 h and than Sco‐Pio 20 group at 24 h post‐shock time (*p* < 0.05 to *p* < 0.01).

**FIGURE 5:**
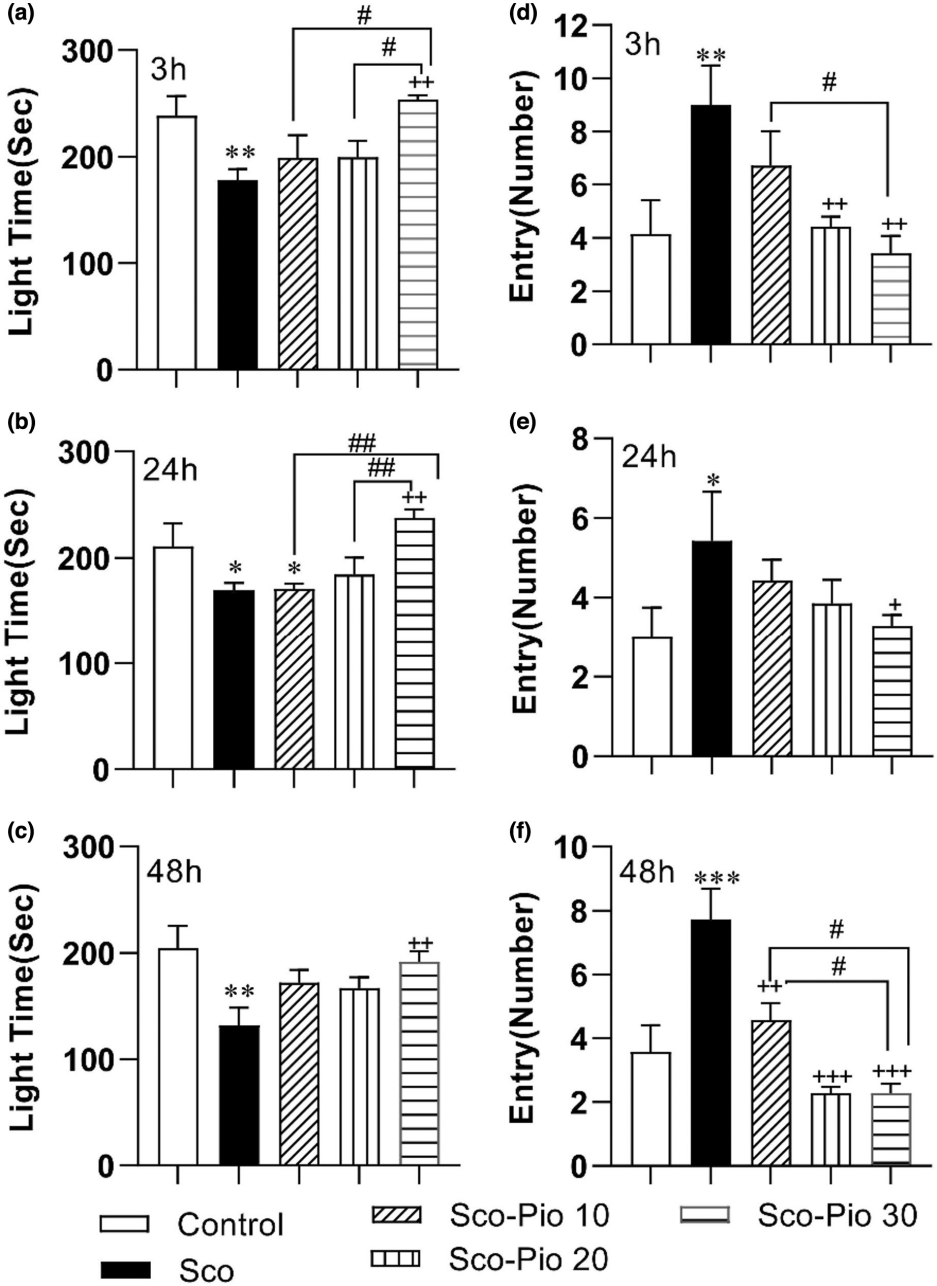
The results of the total light time (a–c) and the number of entries into the light (d–f) in the passive avoidance test. *p < 0.05, **p < 0.01, and ***p < 0.001 present the difference between other groups and Control group, ^+^ *p* < 0.05, ^++^ *p* < 0.01, and ^+++^ *p* < 0.001 present the difference between other groups and the Sco group, ^#^ *p* < 0.05 and ^##^ *p* < 0.01 present the difference between Sco‐Pio 10, Sco‐Pio 20, and Sco‐Pio 30 groups. The data are presented as mean ± SEM (*n* = 7 per group). Sco: scopolamine, Sco‐Pio 10: scopolamine–pioglitazone 10 mg/kg, Sco‐Pio 20: scopolamine–pioglitazone 20 mg/kg, Sco‐Pio 30: scopolamine–pioglitazone 30 mg/kg.

The animals in the Sco group spent a shorter time in the light chamber compared to the animals in the Control group at all times, including 3, 24, and 48 h after the shock (*p* < 0.05 to *p* < 0.01; Figure 4a). The highest dose of Pio (30 mg/kg) was able to increase the stay time in the light compartment at 3, 24, and 48 h after the shock administration (*p* < 0.05 to; Figure 5a) but the medium and lowest doses were not effective. The light time in the Scio‐Pio 30 was longer than that in Sco‐Pio 10 group at 3 and 24 h pot shock time (*p* < 0.05 to *p* < 0.01).

The results of the PA test also showed that the number of entries into the dark segment in the animals of the Sco group was higher than in the Control group at all 3, 24, and 48 h after the shock (*p* < 0.05 to *p* < 0.001). The number of entries into the dark in the Sco‐Pio 20 and Sco‐Pio 30 groups at 3 and 48 h and in the Sco‐Pio 30 group at 24 h after the shock was lower than in the Sco group (*p* < 0.05 to *p* < 0.001). In addition, the dark entries in the Sco‐Pio 30 group were lower than that in Sco‐Pio 10 group at both 3 and 48 h after the shock (*p* < 0.05; Figure 5b).

### 3.3 PPARγ activation attenuated oxidative stress in the hippocampus and cortex of the aged rats

**FIGURE 6:**
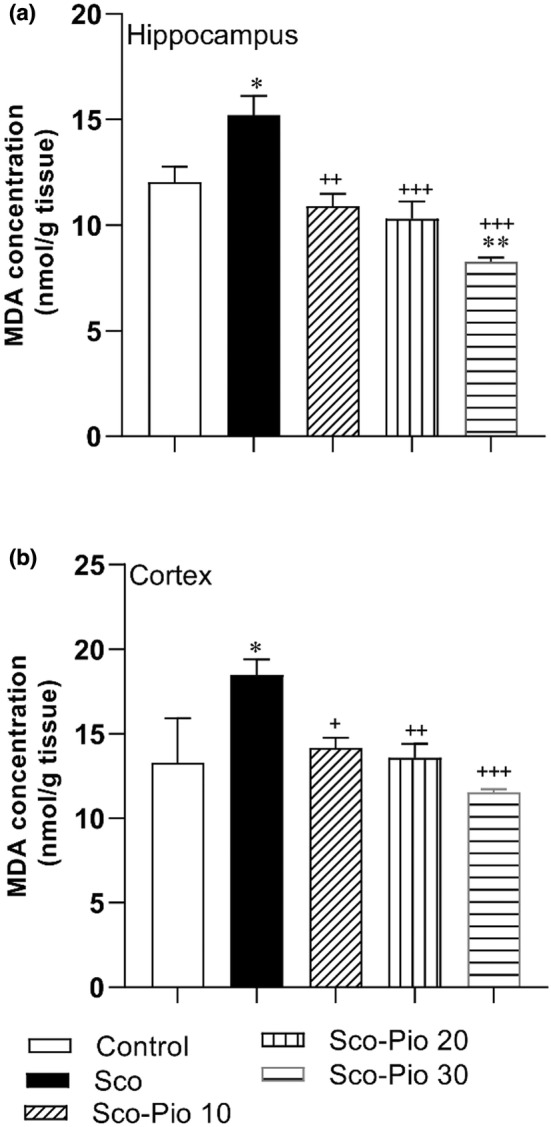
The results of MDA concentration in the hippocampus (a) and cortex (b). *p < 0.05 and **p < 0.01 present the difference between other groups and Control group, ^+^ *p* < 0.05, ^++^ *p* < 0.01, and ^+++^ *p* < 0.001 present the difference between other groups and Sco group. The data are presented as mean ± SEM (*n* = 7 per group). MDA: malondialdehyde, Sco: scopolamine, Sco‐Pio 10: scopolamine–pioglitazone 10 mg/kg, Sco‐Pio 20: scopolamine–pioglitazone 20 mg/kg, Sco‐Pio 30: scopolamine–pioglitazone 30 mg/kg.

MDA concentration was higher in the hippocampus and cortex of the Saco group than in the Control group animals (*p* < 0.05 for both; Figure 5a,b). Pretreatment by all doses of Pio including 10, 20, and 30 mg/kg reversed the effect of MDA and it was shown that MDA level in the hippocampus and cortex of all Sco‐Pio 10, Sco‐Pio 20, and Sco‐Pio 30 groups was lower than that in the Sco group (*p* < 0.05 to *p* < 0.001). There was no significant difference between the effect of three doses of Pio on MDA (Figure 6a,b).

**FIGURE 7:**
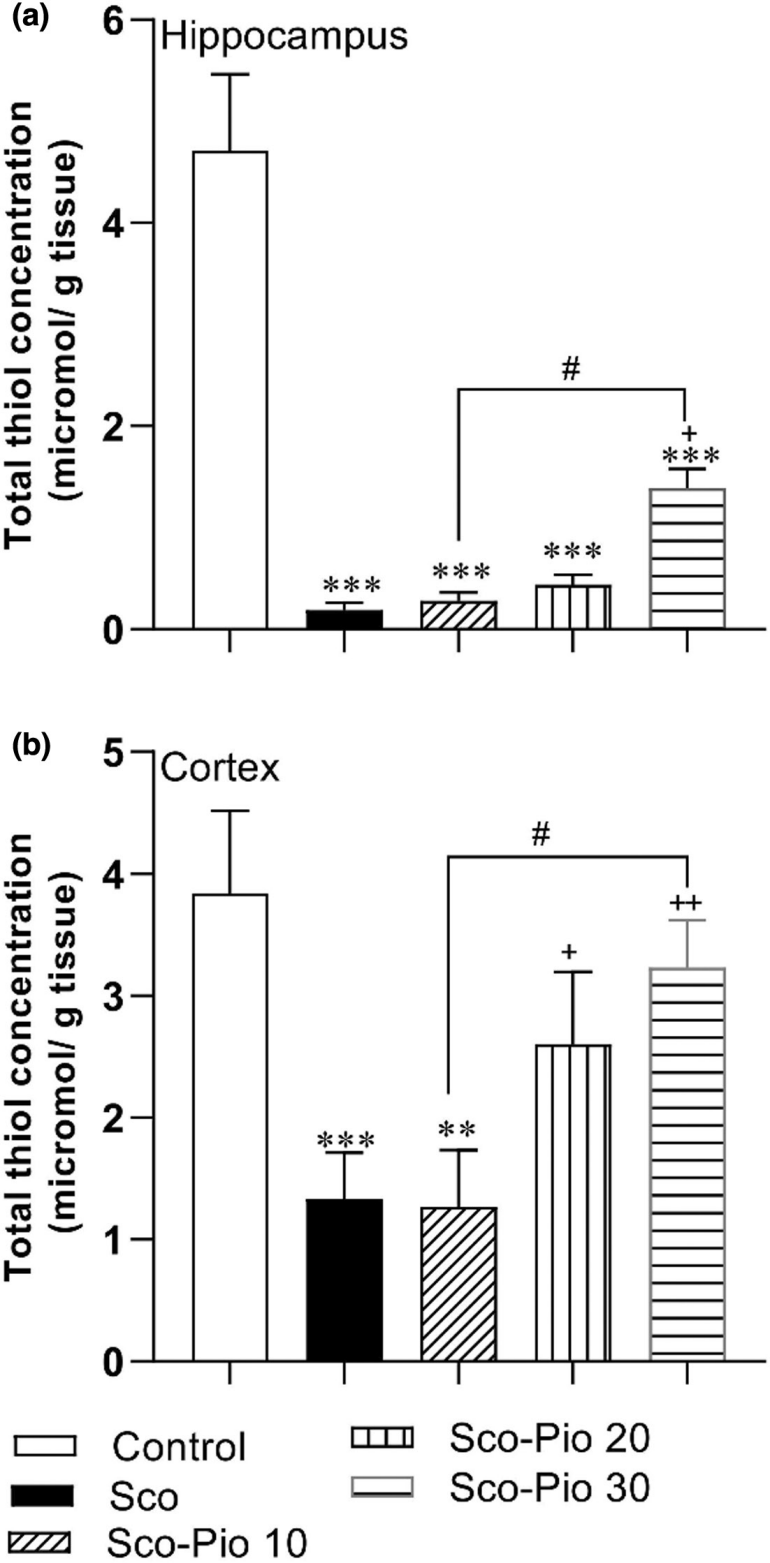
The results of thiol concentration in the hippocampus (a) and cortex (b). **p < 0.01, and ***p < 0.001 present the difference between other groups and Control group, ^+^ *p* < 0.05 and ^++^ *p* < 0.01 present the difference between other groups and Sco group, ^#^ *p* < 0.05 presents the difference between Sco‐Pio 10, Sco‐Pio 20, and Sco‐Pio 30 groups. The data are presented as mean ± SEM (*n* = 7 per group). Sco: scopolamine, Sco‐Pio 10: scopolamine–pioglitazone 10 mg/kg, Sco‐Pio 20: scopolamine–pioglitazone 20 mg/kg, Sco‐Pio 30: scopolamine–pioglitazone 30 mg/kg.

Total thiol concentration in both hippocampus and cortex of Sco was lower than the Control group (*p* < 0.001). The highest dose of Pio increased total thiol content in the hippocampus of the Sco‐Pio 30 group compared to the Sco group (*p* < 0.05). There was no significant difference between Sco‐Pio 10, Sco‐Pio 20, and Control group in the total thiol content in the hippocampus. In addition, total thiol content in the hippocampus of all Sco‐Pio 10, Sco‐Pio 20, and Sco‐Pio 30 groups was lower than the Control group (*p* < 0.001). Both 20 and 30 mg/kg of Pio also improved total thiol content in the cortex of Sco‐Pio 20 and Sco‐Pio 30 groups compared to the Sco group (*p* < 0.05 and *p* < 0.01, respectively) but 10 mg/kg of Pio was not effective (Figure 5a,b). In addition, total thiol content in the hippocampus of the Sco‐Pio 10 group was lower than the Control group (*p* < 0.01). Finally, thiol concentration in both hippocampus and cortex of the Sco‐Pio 20 group was higher than in the Sco‐Pio 10 group (*p* < 0.05) (Figure 7a,b).

**FIGURE 8:**
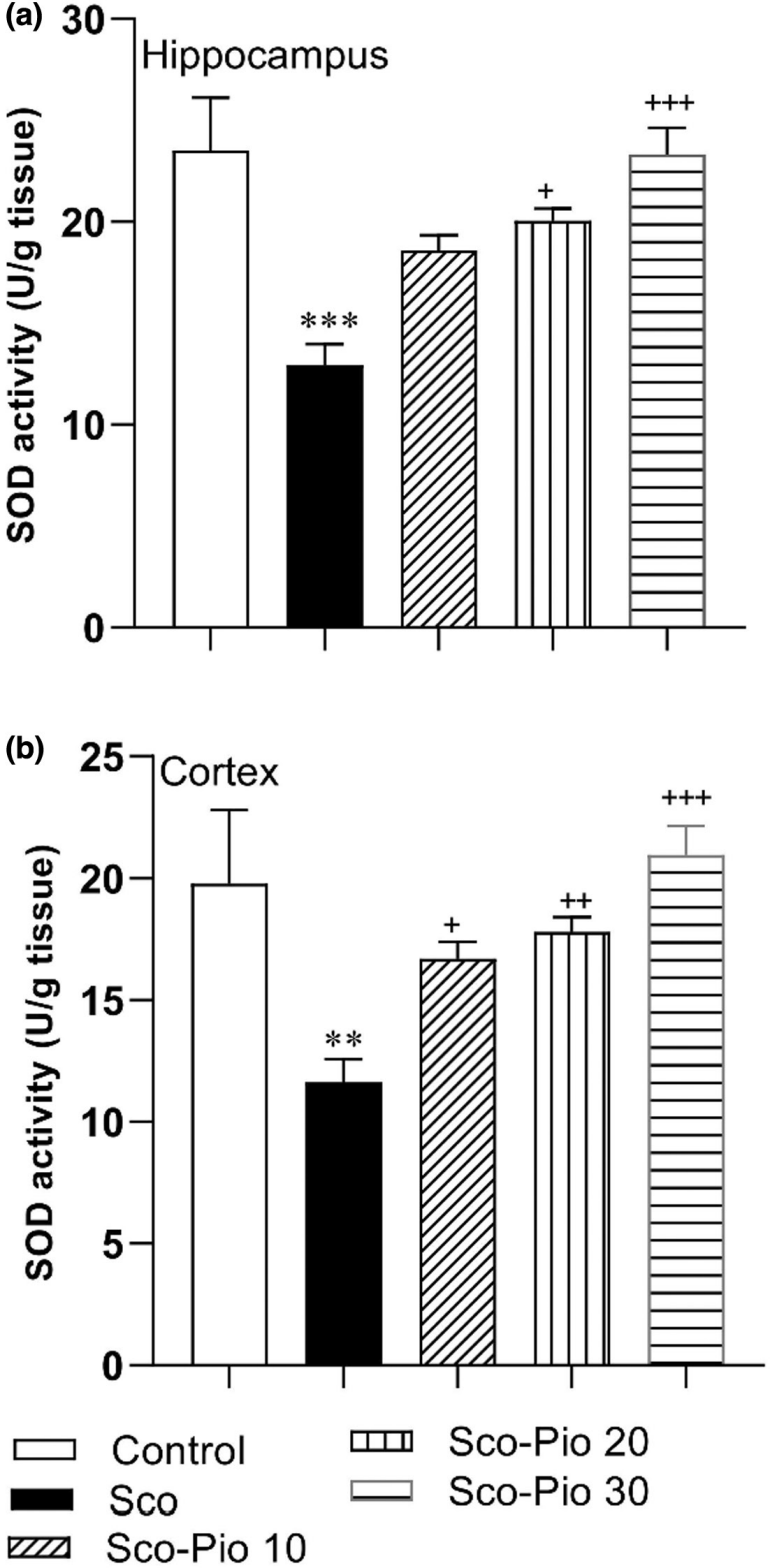
The results of SOD activity in the hippocampus (a) and cortex (b). **p < 0.01, and ***p < 0.001 present the difference between other groups and Control group, ^+^ *p* < 0.05, ^++^ *p* < 0.01, and ^+++^ *p* < 0.001 present the difference between other groups and Sco group. The data are presented as mean ± SEM (*n* = 7 per group). SOD: superoxide dismutase, Sco: scopolamine, Sco‐Pio 10: scopolamine–pioglitazone 10 mg/kg, Sco‐Pio 20: scopolamine–pioglitazone 20 mg/kg, Sco‐Pio 30: scopolamine–pioglitazone 30 mg/kg.

The results of the study showed that both hippocampal and cortical SOD activity was decreased in the Sco group compared to the Control group (*p* < 0.01 and *p* < 0.001, respectively). The results also showed that SOD activity in the hippocampus of Sco‐Pio 20 and Sco‐Pio 30 groups and the cortex of all Sco‐Pio 10, Sco‐Pio 20, and Sco‐Pio 30 groups was higher than that in the Sco group (*p* < 0.05 to *p* < 0.001) (Figure 8a,b).

**FIGURE 9:**
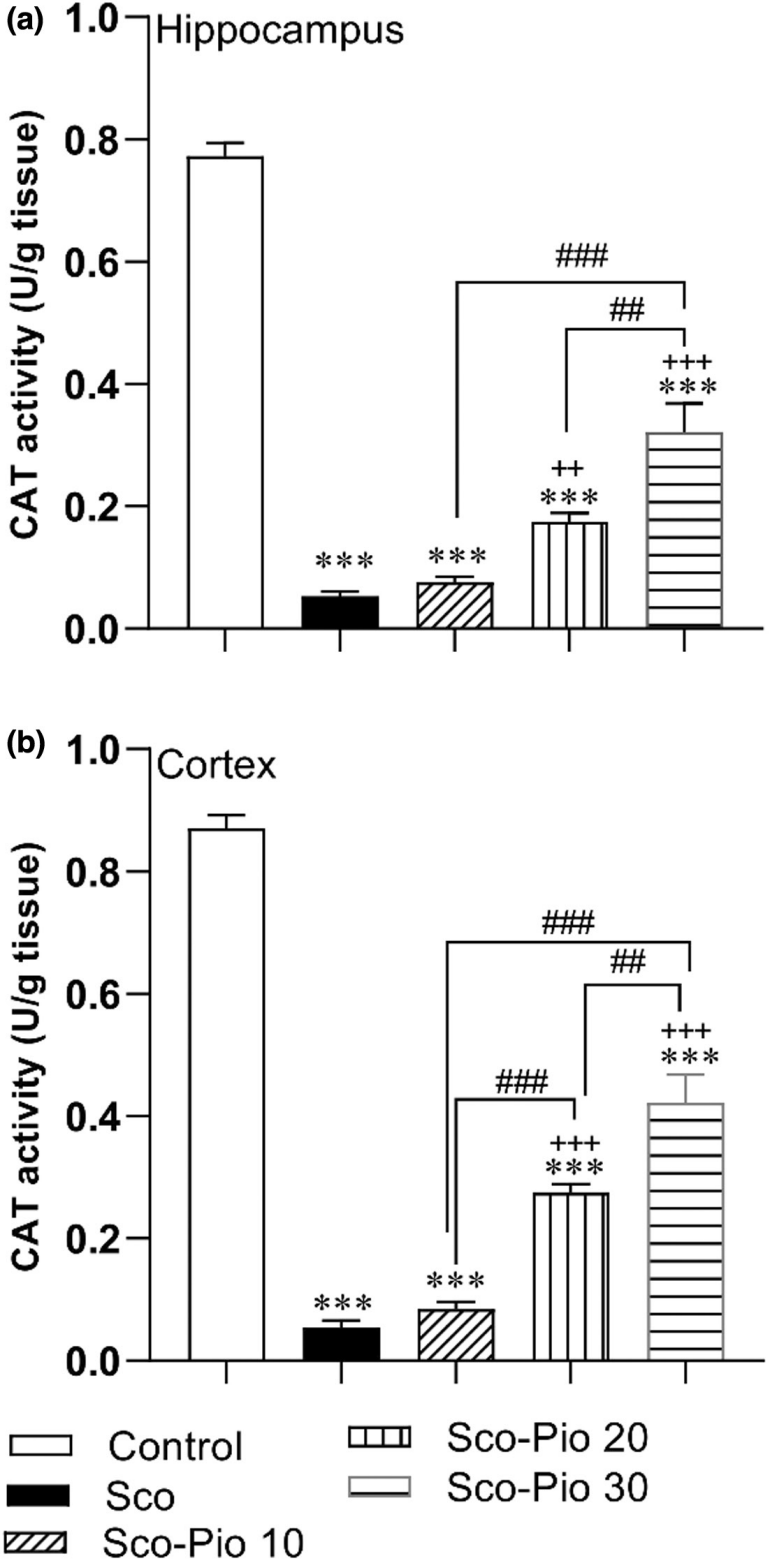
The results of CAT activity in the hippocampus (a) and cortex (b). ***p < 0.001 presents the difference between other groups and Control group, ^++^ *p* < 0.01 and ^+++^ *p* < 0.001 present the difference between other groups and the Sco group, ^##^ *p* < 0.01 and ^###^ *p* < 0.001 present the difference between Sco‐Pio 10, Sco‐Pio 20, and Sco‐Pio 30 groups. The data are presented as mean ± SEM (*n* = 7 per group). CAT: catalase, Sco: scopolamine, Sco‐Pio 10: scopolamine–pioglitazone 10 mg/kg, Sco‐Pio 20: scopolamine–pioglitazone 20 mg/kg, Sco‐Pio 30: scopolamine–pioglitazone 30 mg/kg.

The biochemical results also showed that CAT activity in both hippocampus and cortex of the Sco group was decreased compared to the Control group (*p* < 0.001 for both). Pretreatment by 20 and 30 mg/kg Pio improved CAT activity in both hippocampus and cortex of both Sco‐Pio 20 and Sco‐Pio 30 groups compared to the Sco group (*p* < 0.01 to *p* < 0.001). Both hippocampal and cortical CAT in Sco‐Pio 30 group was higher than that of both Sco‐Pio 10 and Sco‐Pio 20 groups (*p* < 0.01 to *p* < 0.001). In addition, CAT activity in both hippocampus and cortex of all Sco‐Pio 10, Sco‐Pio 20, and Sco‐Pio 30 groups was lower than that in the Control group (*p* < 0.001) (Figure 9a,b).

## 4 DISCUSSION

The results of the present study showed that Pio as an agonist of PPARγ improved the learning and memory of Sco‐injected aged rats. It also attenuated oxidative stress in the hippocampus and cortex.

Aging is a complex biochemical phenomenon that can contribute to the functional deterioration of different body tissues. In addition, it increases the vulnerability to many chronic diseases (Ma et al., 2020). Cholinergic dysfunction has been repeatedly suggested to be affected by aging to contribute to cognitive impairments (Pepeu & Marconcini Pepeu, 1994). In the current research, Sco‐treated rats had weaker performances in the MWM which was indicated by longer time and distance for reaching the platform during 5 days of learning of MWM in the Sco group compared to the Control group. The rats of the Sco group also spent a shorter time and traveled a shorter distance in the target area in the probe test of MWM. These data confirm that Sco administration impaired spatial learning and memory of the aged rats. These results were also confirmed by the results of the PA test in which the rats of the Sco group had a shorter delay for entering the dark and spent a longer time in the dark than the animals of the Control group. The rats of the Sco group also spent a shorter time in the light segment of the PA test and had a higher number of entries into the dark chamber than the Control group.

The possible molecular pathways involved in cognitive deficits due to aging have not been sufficiently understood so far. Oxidative stress has been shown to play a key role in the progression of aging (Melo et al., 2011). Brain tissue oxidative damage accompanied by cholinergic dysfunction has been repeatedly considered to have important roles in cognitive deficits and learning and memory disabilities, especially during aging (Giudetti et al., 2018). In the current study, the poorer performance of the Sco‐ treated rats in MWM and PA tests was accompanied by a higher concentration of MDA as an index of lipid peroxidation, lower level of thiol, and lower activities of SOD and CAT which may imply that oxidative damage in the hippocampus and cortex has an important role learning and memory impairment.

In support of this idea, aging has been documented to induce neurodegeneration (Zhang et al., 2008), alteration of neurotransmission in different areas of the brain (Wang et al., 2018), and oxidative stress (Zhang et al., 2008). Other studies have shown that Sco‐induced memory impairment has been linked to increased oxidative stress in the brain, as well as specific regions associated with learning and memory (Budzynska et al., 2015). We also previously showed Sco‐ induced learning and memory impairment was accompanied by oxidative damage in the hippocampus and cortex which was improved by the antioxidant agents (Hejazian et al., 2016; Hosseini et al., 2015).

Activating PPAR‐γ by other agonists has been frequently examined to treat oxidative stress‐related diseases such as diabetes (Chan et al., 2010; Kleinhenz et al., 2009; Yousefipour et al., 2010) and some brain disease such as AD and Parkinson's disease (Hunter & Bing, 2007; Lee et al., 2010). It has also been frequently shown that PPAR‐γ agonists including Pio was able to decrease the superoxide anion generation and lipid peroxidation while they were able to increase the activities of antioxidant enzymes such as SOD and CAT (Girnun et al., 2002; Villegas et al., 2004; Yoo et al., 1999). With keeping in mind that the antioxidants can reverse learning and memory deficits induced by Sco we assumed that Pio may improve learning and memory impairments. Interestingly, pretreatment of the Sco‐treated aged rats with Pio increased the latency to enter the dark while, decreasing the total time spent in the dark chamber, increasing the total time spent in the light, and decreasing entries into the dark indicating improvement in learning and memory. Also in MWM, the rats that received Pio have a better function to find the platform in 5 days and they had a shorter traveling time and distance to reach the platform. They also did better tasks on probe day and traveled longer time and distance in the target area than the Sco group. The results were in agreement with our previous works in which Pio improved learning and memory impairments induced by lipopolysaccharide (Beheshti et al., 2019). In our previous studies, the beneficial effects of Pio were attributed to antioxidative effects (Beheshti et al., 2019). Other studies also confirmed the learning and memory improving effects of Pio in other animal models and its beneficial effects on cognitive functions including learning and memory were attributed to the antioxidant effects (McGuire et al., 2019).

**FIGURE 10:**
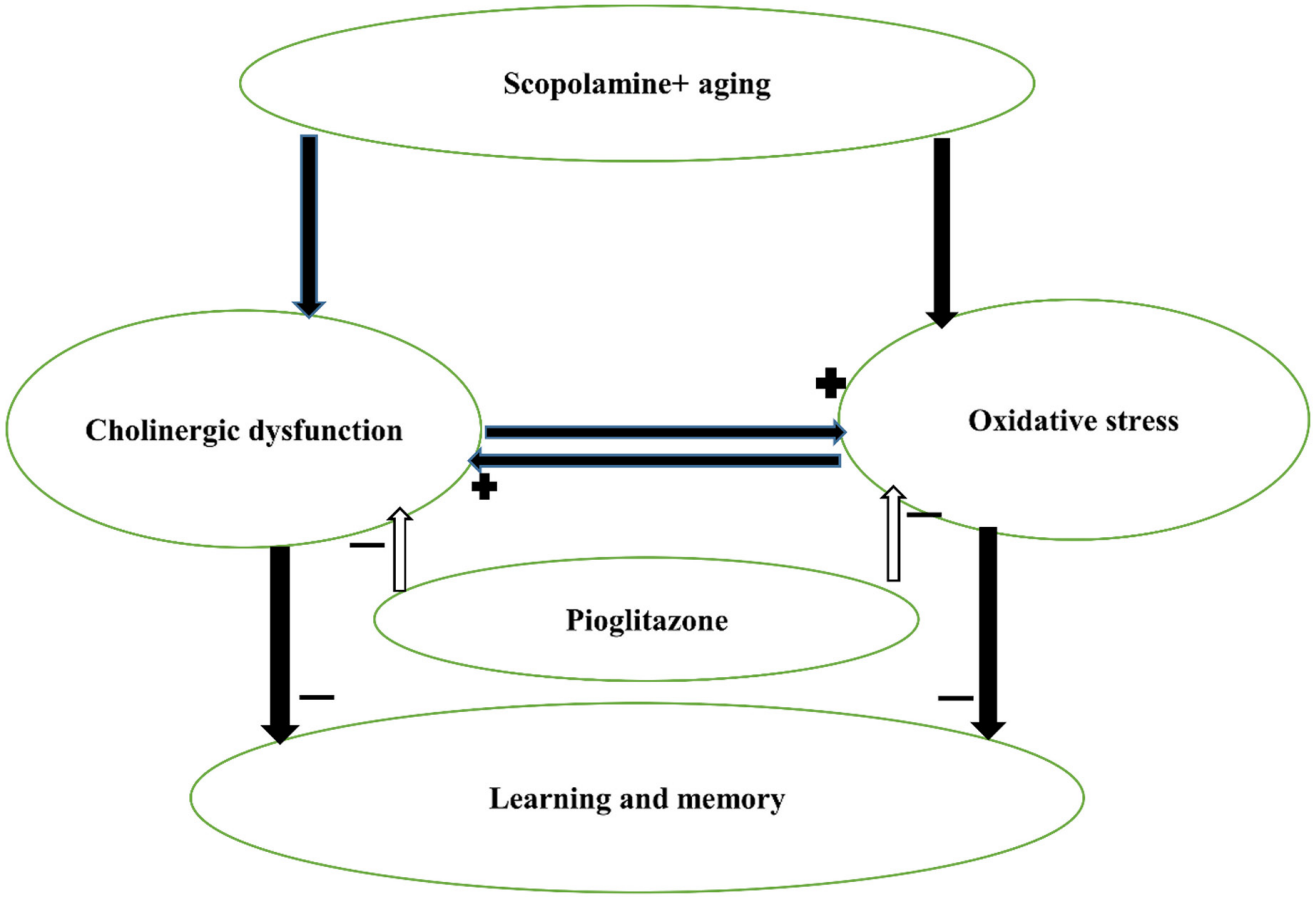
A graphical diagram that illustrates the main findings of this study and their connection.

Other recent studies have suggested that PPARγ agonists have some benefits for the nervous system (62–64). In particular, Pio supplementation has been capable of protecting cultured hippocampal neurons against neurotoxic agents (65). Additionally, ROS production and brain tissue oxidative damage have been well known to play an important role in learning and memory impairments (Beheshti, Hosseini, et al., 2017). Also previously we showed that Pio had some antioxidant effects (Dobrian et al., 2004). The results of the current study also showed that Pio decreased MDA while increasing thiol, SOD, and catalase. Thus, the ability of Pio to reverse Sco‐induced learning and memory impairments in aged rats which were seen in the present study may at least in part be due to enhancement of the antioxidant defense system and attenuation of oxidative stress in the hippocampus and cortex (Pathan et al., 2006). This action might result from its ability to overcome the pro‐oxidant effects of Sco in the hippocampus and cortex, through the increase in antioxidant defense systems including GSH, SOD, and CAT, and a decrease in MDA and nitrite levels in the hippocampus and cortex (Ciobica et al., 2011). Based on the results obtained from the behavioral and biochemical studies, it may be suggested that Pio may act directly as a free radical scavenger or regulator to ameliorate oxidative stress in the nervous system (Ekladious & El Sayed, 2019). A graphical diagram illustrates the main findings of this study and their connection (Figure 10).

## 5 CONCLUSION

In conclusion, the results of the current study showed that PPARγ activation by Pio as an agonist improved learning and memory in aged rats. It seems that the effect is due to its ability in attenuating oxidative stress in the hippocampus and cortex.

## AUTHOR CONTRIBUTIONS

Farimah Beheshti: Visualization, Data collection, Writing original draft; Masoumeh Gholami: Data collection, Visualization, Writing an original draft; Zahra Ghane: Data collection, Behavioral tests; Seyedeh Elnaz Nazari: Data collection, Behavioral tests; Maryam Salari: Data collection, Behavioral tests; Sadegh Shabab: Data Collection, Behavioral tests; Mahmoud Hosseini: Conceptualization, Methodology, Validation, Resources, Data curation, Writing, Review and editing, Supervision, Project administration, Funding acquisition.

## CONFLICT OF INTEREST

The authors have no conflict of interest to declare
